# *Lactobacillus delbrueckii* Interfere With Bile Acid Enterohepatic Circulation to Regulate Cholesterol Metabolism of Growing–Finishing Pigs via Its Bile Salt Hydrolase Activity

**DOI:** 10.3389/fnut.2020.617676

**Published:** 2020-12-11

**Authors:** Gaifeng Hou, Wei Peng, Liangkai Wei, Rui Li, Yong Yuan, Xingguo Huang, Yulong Yin

**Affiliations:** ^1^College of Animal Science and Technology, Hunan Agricultural University, Changsha, China; ^2^Hunan Co-Innovation Center of Animal Production Safety, Changsha, China; ^3^Key Laboratory of Agro-ecological Processes in Subtropical Region, Scientific Observing and Experimental Station of Animal Nutrition and Feed Science in South-Central, Ministry of Agriculture, Hunan Provincial Engineering Research Center for Healthy Livestock and Poultry Production, Institute of Subtropical Agriculture, Chinese Academy of Sciences, Changsha, China

**Keywords:** *Lactobacillus delbrueckii*, ileal microbiota, cholesterol, bile acids, enterohepatic circulation, pigs

## Abstract

Microbiota-targeted therapies for hypercholesterolemia get more and more attention and are recognized as an effective strategy for preventing and treating cardiovascular disease. The experiment was conducted to investigate the cholesterol-lowering mechanism of *Lactobacillus delbrueckii* in a pig model. Twelve barrows (38.70 ± 5.33 kg) were randomly allocated to two groups and fed corn–soybean meal diets with either 0% (Con) or 0.1% *Lactobacillus delbrueckii* (Con + LD) for 28 days. *L. delbrueckii–*fed pigs had lower serum contents of total cholesterol (TC), total bile acids (TBAs), and triglyceride, but higher fecal TC and TBA excretion. *L. delbrueckii* treatment increased ileal *Lactobacillus* abundance and bile acid (BA) deconjugation and affected serum and hepatic BA composition. Dietary *L. delbrueckii* downregulated the gene expression of ileal apical sodium-dependent bile acid transporter (*ASBT*) and ileal bile acid binding protein (*IBABP*), and hepatic farnesoid X receptor (*FXR*), fibroblast growth factor (*FGF19*), and small heterodimer partner (*SHP*), but upregulated hepatic high-density lipoprotein receptor (*HDLR*), low-density lipoprotein receptor (*LDLR*), sterol regulatory element binding protein-2 (*SREBP-2*), and cholesterol-7α hydroxylase (*CYP7A1*) expression. Our results provided *in vivo* evidence that *L. delbrueckii* promote ileal BA deconjugation with subsequent fecal TC and TBA extraction by modifying ileal microbiota composition and induce hepatic BA neosynthesis via regulating gut–liver FXR–FGF19 axis.

## Introduction

Cholesterol is an indispensable fundamental building block for all cell membranes, but long-term high level of blood cholesterol may induce hypercholesterolemia-associated cardiovascular diseases (CVDs), a major contributing factor of adult deaths worldwide (1, 2). It is reported that a 1% reduction in blood cholesterol translates to a 2% decrease in heart disease risk (3, 4). Blood cholesterol level is determined by dietary fat and cholesterol intake and the body's cholesterol biosynthesis and excretion (5). Endogenous synthetic cholesterol accounts for nearly 70%, whereas the remaining 30% amount is mainly derived from animal products (6). Pork products are rich in cholesterol ranging from 57 mg/100 g in loin to 116 mg/100 g in dewlap (7). In China, pork is the most popular animal meat, and its production and consumption contribute about 50% of global pork output ranking first in the world (8). Therefore, clarification of underlying mechanisms of cholesterol metabolism in pigs and development of low cholesterol pork products has a promising potential of scientific researches and consumer markets.

Cholesterol is a precursor to bile acid (BA) biosynthesis. Approximately 30% to 40% of cholesterol is converted into primary BAs in liver via two pathways, with CYP7A1 as the rate-limiting enzyme in the classic pathway and CYP7B1 as an important enzyme in the alternative pathway (9, 10). Synthesized primary BAs are conjugated either with taurine or glycine and temporarily stored in the gallbladder. Upon cholecystokinin stimulation, often as a result of a meal, BAs are released into the duodenum via the bile duct. About 95% of BAs are reabsorbed all along the intestine, especially in the distal ileum, via passive diffusion and carrier-mediated transports entering enterohepatic cycle to maintain the BA pool homeostasis (11, 12). In each cycle, nearly 4% BAs are excreted along with feces, which is offset by the hepatic *de novo* synthesis of BAs from cholesterol (12). Obviously, the conversion of cholesterol to the BAs is the major route for cholesterol excretion, and the increased fecal BA excretion favors the conversion from cholesterol to BAs and reduces its release into the systemic circulation (13).

The hypocholesterolemic effect of *Lactobacillus* or its related products are reported extensively in animals and clinical researches (4, 12, 14, 15). Several proposed potential cholesterol-lowering mechanisms of *Lactobacillus* products chiefly cover cholesterol assimilation, cholesterol conversion to coprostanol, BSH activity, production of short fatty acids, and regulation of key enzyme in cholesterol metabolism (3, 12). However, the majority of explanations were based on *in vitro* test or high-fat or cholesterol animal models, and there was no adequate supporting evidence from normal subjects to validate these assumptions. Interestingly, our prior work confirmed that dietary *Lactobacillus delbrueckii* [1.01 × 10^9^ colony-forming units (CFU)/g] lowered serum TC and triglyceride (TG) and increased the fecal TC and total BA (TBA) excretion of fatten pigs in commercial condition; unfortunately, we did not explore the further mechanism (16).

Given that the close relationship between cholesterol and BA metabolism, we supposed that *L. delbrueckii* with BSH activity affected the enterohepatic circulation of BA, which contributed to the reduced serum TC in a pig model. Therefore, we investigated the BSH activity of *L. delbrueckii* through plate assay and gene identification and also evaluated the effects of *L. delbrueckii on* intestinal microbiota, BA and cholesterol metabolism, and tissue lipids of growing–finishing pigs.

## Materials and Methods

All protocols and procedures involved in the experiment were approved by the Animal Ethics Committee of Hunan Agricultural University (Changsha, China). *L. delbrueckii* was provided by the microbiology functional laboratory of the College of Animal Science and Technology in the Hunan Agricultural University (Changsha, China). The strain was activated and sent to the PERFLY-BIO (Changsha, China) for large-scale production, and the viable count of final products reached 5 × 10^11^ CFU/g.

### Animals and Experimental Design

Twelve Landrace × Yorkshire crossbred barrows with an average initial body weight of 38.70 ± 5.33 kg were randomly allocated to two groups, and each group had six pigs individually housed in the metabolism cage. Pigs were fed with corn–soybean meal diets (basal diets, Con) or basal diets containing 0.1% *L. delbrueckii* preparation (5 × 10^10^ CFU/g, Con + LD) for 28 days. The basal diets (Table 1) were formulated to meet the nutritional requirement of 50- to 75-kg pigs recommended by the NRC 2012 (17). All pigs were fed twice each day (8:00 A.M. and 3:00 P.M.) and had free access to water. The body weight of each pig was weighed at the beginning and end of the experiment, and the daily feed consumption per pig was recorded during the experimental period. Fecal samples were collected, freeze-dried, and stored at −20°C for total cholesterol (TC) and TBA detection. On day 29, the jugular vein blood samples were collected from the fasting pigs before slaughter using electrical stunning. Serum was obtained, aliquoted, and stored at −20°C for lipid analysis and BA profiles quantification. Digesta (in ileum) and tissues (in ileum, liver, longissimus dorsi, subcutaneous fat, and leaf lard) were quickly removed, snap-frozen in the liquid nitrogen, and stored at −80°C for microbiota composition, BA quantification, gene mRNA expression, lipid profile, and enzyme activity measurements.

**Table 1:** Diet composition and nutritional levels of basal diets (air-dry basis, %).

**Ingredients**	**Contents**
Corn	66.76
Wheat middling	4.00
Wheat bran	6.00
Soybean meal (43% crude protein)	18.00
Soybean oil	1.00
L-Lysine	0.24
Premix^a^	4.00
Total	100.00
**Calculated nutritional levels**	
Digestible energy (DE, kcal/kg)	3,413.79
Crude protein	14.82
Standardized ileal digestible lysine (SID Lys)	0.85
Calcium	0.60
Total phosphorus	0.55

a *The premix provided the following per kg of diet:VA2 512 IU, VD3 1 200 IU, VE 34 IU, VK3 1.5 mg, VB12 17.6 μg, lactoflavin 2.0.5 mg, pantothenic acid 6.8 mg, niacin 20.3 mg, choline chloride 351, Mn 10 mg, Fe 50 mg, Zn 50 mg, Cu 20 mg, I 0.3 mg, Se 0.3 mg*.

### Qualitative Determination of BSH Activity

Qualitative BSH activity of *L. delbrueckii* was measured according to the method introduced by Jayashree et al. (18) and Guo et al. (19) with a minor modification. Briefly, five sterile paper discs (8-mm diameter) were placed on an MRS agar plate containing 2 g/L taurodeoxycholate and glycodeoxycholate, 2 g/L sodium thioglycolate and 0.37 g/L CaCl_2_, and 100 μL *L. delbrueckii* solution (1 g bacterial power was diluted with 9 mL of sterile water to get final concentrations of 1.5 × 10^10^ CFU/mL) were added to the paper discs immediately. The plates were incubated at 37°C for 72 h. The BA precipitates (i.e., opaque granular white colonies with silvery shine) around the discs were considered as BSH activity.

Genomic DNA of the *L. delbrueckii* was extracted using the TIANamp Stool DNA kit [Tiangen Biotech (Beijing) Co., Ltd, China]. According to the report by Jayashree et al. (18), two primers (Table 2) for BSH1 and BSH2 were used to amplify the corresponding target gene, and the polymerase chain reaction (PCR) product sizes were 927 and 978 bp, respectively. The PCR reactions were carried out in 25-μL reaction system in a TaKaRa PCR Thermal Cycler. The PCR conditions were 5 min at 94°C for the initial denaturation followed by 35 cycles of denaturation at 94°C for 30 s, 1 min at 52°C for annealing, 1 min at 72°C for extension, and 5 min at 72°C for the final extension.

**Table 2:** Primers used in the study.

**Items**	**Gene**	**Sequence (5**′**-3**′**)**
BSH gene	*BSH1*	F: GCCACCATGGTAATGTGCACGGCCGTTTCC
		R: CGATGGATCCTTAGGGTACTTGCGATAGG
	*BSH2*	F:ACCCATGGGTATGTGCACGAGCATCAACGTCA
		R: AAGGATCCGTTCAATTTCACCGGCGCCCAA
BA receptor and signaling	*FXR*	F: GGTCCTCGTAGAATTCACAA
		R: TGAACGGAGAAACATAGCTT
	*FGF19*	AGTACTCGGATGAGGACTGTGCTT
		AGAGACGGGCAGATGGTGTTTCTT
	*SHP*	F: GCCTACCTGAAAGGGACCAT
		R: CAACGGGTGTCAAGCCTTTA
BA transport	*ASBT*	F: TACGCGGTATACAGGAAATGGTA
		R: TTTGCCTTTTGGAATGATGACT
	*IBABP*	F: GTGAACAGCCCCAACTACCACCA
		R: TCGTAGCTCACGCCTCCGAC
BA biosynthesis	*CYP7A1*	F: GAAAGAGAGACCACATCTCGG
		R: GAATGGTGTTGGCTTGCGAT
	*CYP27A1*	F: ACTGAAGACCGCGATGAAAC
		R: CAAAGGCGAATCAGGAAGGG
Cholesterol biosynthesis and transport	*SREBP-2*	F: GATGGGCAGCAGAGTTCC
		R: ACAGCAGCAGGTCACAGGT
	*HMGR*	F: ATGGCATGACTCCAGTGGTACGTT
		R: GCAAATCTGCTGGTGCTGTCGAAT
	*HDLR*	F: CACTATGCCCAGTACGTGCTC
		R: CCTGAATGGCCTCCTTATCCTT
	*LDLR*	F: TTCTTCACCAACCGCCACGAG
		R: CTCAGTGTCCAGAGCGACC
Housekeeping gene	*GAPDH*	F: ATGGTGAAGGTCGGAGTGAAC
		R: CTCGCTCCTGGAAGATGGT

*BSH, bile salt hydrolase; FXR, farnesoid X receptor; FGF19, fibroblast growth factor; SHP, small heterodimer partner; ASBT, apical sodium-dependent bile acid transporter; IBABP, ileal bile acid binding protein; CYP7A1, Cholesterol-7α hydroxylase; CYP27A1, cholesterol-27α hydroxylase; SREBP-2, sterol regulatory element binding protein-2; HMGR, 3-hydroxy-3-methyl glutaryl coenzyme A reductase; HDLR, high-density lipoprotein receptor; LDLR, low-density lipoprotein receptor*.

### Determination of Serum and Tissue Lipids

Fasting blood of pigs were collected and placed at room temperature for 30 min, and the serum were separated by centrifugation (3,000 revolutions/min for 10 min at 4°C). Serum concentrations of TG, glucose (GLU), TBA, TC, high-density lipoprotein cholesterol (HDL-C), and high-density lipoprotein cholesterol (LDL-C) were measured by the BS 200 automatic blood biochemical analyzer (Mindray) with corresponding kits.

The total protein contents (g protein/L) in tissues were quantified using a BCA protein assay reagent kits (Nanjing Jiancheng Bioengineering Institute, Nanjing, China). About 100 mg of liver, longissimus dorsi, subcutaneous fat, or leaf lard was homogenized with 1 mL of chloroform/methannol solution (2:1, vol/vol), respectively. The homogenate were centrifuged at 3,000 revolutions/min for 10 min at 4°C to extract tissue lipids. The contents of TC (mmol/g · protein), TG (mmol/g · protein), and TBA (μmol/g · protein) in the selected tissue were measured by corresponding commercial kits (Nanjing Jianchen Bioengineering Institute, Jiangsu, China).

### Measurement of Hepatic Enzyme Activity Using ELISA Kits

Hepatic total protein contents (g protein/L) were measured as described above, and the concentrations of hepatic 3-hydroxy-3-methyl glutaryl coenzyme A reductase (HMGR, U/g · protein) and cholesterol-7α hydroxylase (CYP7A1, U/g · protein) were measured following the instruction of corresponding commercial ELISA Kits (Jiangsu Yutong Biological Technology Co., Ltd., Jiangsu).

### Fecal TC and TBA Excretion

Fecal lipids were extracted as described above for TC analysis. Fecal TBA was extracted according to the method by De Smet et al. (5). Briefly, 1 g frozen fecal sample was dissolved in 40 mL methanol. After 4-min sonication and 1-h shock, the mixture was centrifuged at 10,000 *g* for 10 min to collect the supernatants. Total TC (mmol/L) and TBA (μmol/L) concentrations in the supernatants were determined using a commercial kit purchased from the Nanjing Jianchen Bioengineering Institute. At last, fecal TC and TBA contents (mg/g) were obtained by formula conversion.

### BA Profile Analysis

#### Metabolite Extraction

About 30 mg of solid samples (ileal digesta or hepatic tissue) were homogenized in 100 μL of precooled ultrapure water, vortexed with 5,000 μL of iced methanol and 10 μL of internal standard solution (for liquid sample, 100 μL serum was directly vortexed with 500 μL of iced methanol and 10 μL of internal standard solution), incubated at −20°C for 20 min for depositing protein, and centrifuged at 14,000 relative centrifugal force (rcf)/min for 15 min at 4°C. The supernatants were vacuum dried for subsequent analysis.

#### Ultraperformance Liquid Chromatography–Mass Spectrometry (UPLC-MS) Analysis

BA profiles were analyzed with a Waters ACQUITY UPLC I-Class coupled with a 5500 QTRAP mass spectrometer with an ESI source (Waters, Milford, MA). Briefly, the samples above were resolved in 1:1 (vol/vol) methanol solution and centrifuged at 14,000 rcf/min for 15 min at 4°C to obtain supernatants. The supernatants were separated using an ACQUITY UPLC BEH C18 chromatographic column (1.7 μm, 100 × 2.1 mm) (Waters, Milford, MA), and column temperature reached 50°C. The injection volume was 2 μL. A mobile phase system included Solvent A (0.1% FA solution) and Solvent B (methanol), in a gradient system at a flow rate of 0.3 mL/min. The mobile phase B was linearly changed as follows: from 60 to 65% (0–6 min), 65 to 80% (6–13 min), 65 to 80% (6–13 min), 80 to 90% (13–13.5 min), and stabilization at 90% (13.5–15 min). The mass spectrometer was used in Multiple Reaction Monitoring function in the ESI-negative mode to achieve information of tested ion pairs. The operating parameters were as follows: source temperature 550°C; ion source gas1 55 psi; ion source gas1 55 psi; curtain gas 40 psi; and IonSapary voltage floating −4,500 V. UPLC-MS raw data were analyzed using Multiquant™ software (v. 2.1) to obtain calibration equations and the quantitative concentrations of each BA.

### Ileal Microbiota Analysis

Microbiota composition was analyzed according to our previous study (20, 21). Briefly, the ileal digesta were collected, frozen in liquid nitrogen, and stored at −80°C for further analysis. Total DNA was extracted and purified from digesta samples (*n* = 5 pigs/group) using TIANamp Stool DNA kit [Tiangen Biotech (Beijing) Co., Ltd, China]. DNA quality and quantity were evaluated by gel electrophoresis and a NanoDrop ND-1000 spectrophotometer (Thermo Fisher Scientific, USA), respectively. Ten acceptable DNA samples were delivered to Novogene (Beijing) for 16S rDNA sequencing.

The V3–V4 hypervariable region of the bacterial 16S rDNA gene was amplified with the barcoded universal primers (341F-806R). Purified amplicons were sequenced on the Illumina HiSeq platform (Illumina, USA) according to the standard procedures in Novogene (Beijing). Sequences with 97% similarity were assigned to the same operational taxonomic units (OTUs). An OTU table was further generated to record the abundance of each OTU in each sample, and a profiling histogram was made using R software (v. 3.1.1) to represent the relative abundance of taxonomic groups from phylum to species. A Venn diagram was generated to visualize the occurrence of shared and unique OTUs among groups.

### Real-Time PCR

Total RNA of ileal or hepatic tissue was isolated and reversed transcribed to cDNA as previously described (20, 21). The two-step qRT-PCR reactions were performed in triplicate on 96-well plates using a 7500 Real-time PCR system (Applied Biosystems, Foster, CA) with the SYBR Premix Ex Taq™ (TaKaRa Biotechnology (Dalian), China). The primer sequences (Table 2) of farnesoid X receptor (*FXR*), fibroblast growth factor (*FGF19*), *SHP, ASBT, IBABP, CYP7A1*, cholesterol-27α hydroxylase (*CYP27A1*), sterol regulatory element binding protein-2 (*SREBP-2*), *HMGR*, high-density lipoprotein receptor (*HDLR*), low-density lipoprotein receptor (*LDLR*) and *GAPDH* were synthesized by the Sangon Biotech (Shanghai, China). Target gene expression was calculated by the 2^−ΔΔt^ method relative to *GAPDH* gene amplification.

### Statistical Analysis

All results were expressed as mean ± SD. Statistical analyses, except for microbiota data, were conducted by the two-tailed unpaired Student *t-*test of SPSS 17.0 (SPSS Inc., Chicago, IL, USA), with individual pig as an experimental unit. The Kruskal test was used for *post hoc* comparison of taxonomy. For all tests, *P* < 0.05 was considered as significant difference, while 0.05 < *P* < 0.10 as a tendency.

## Results

### Qualitative Identification of BSH Activity

**Figure 1:**
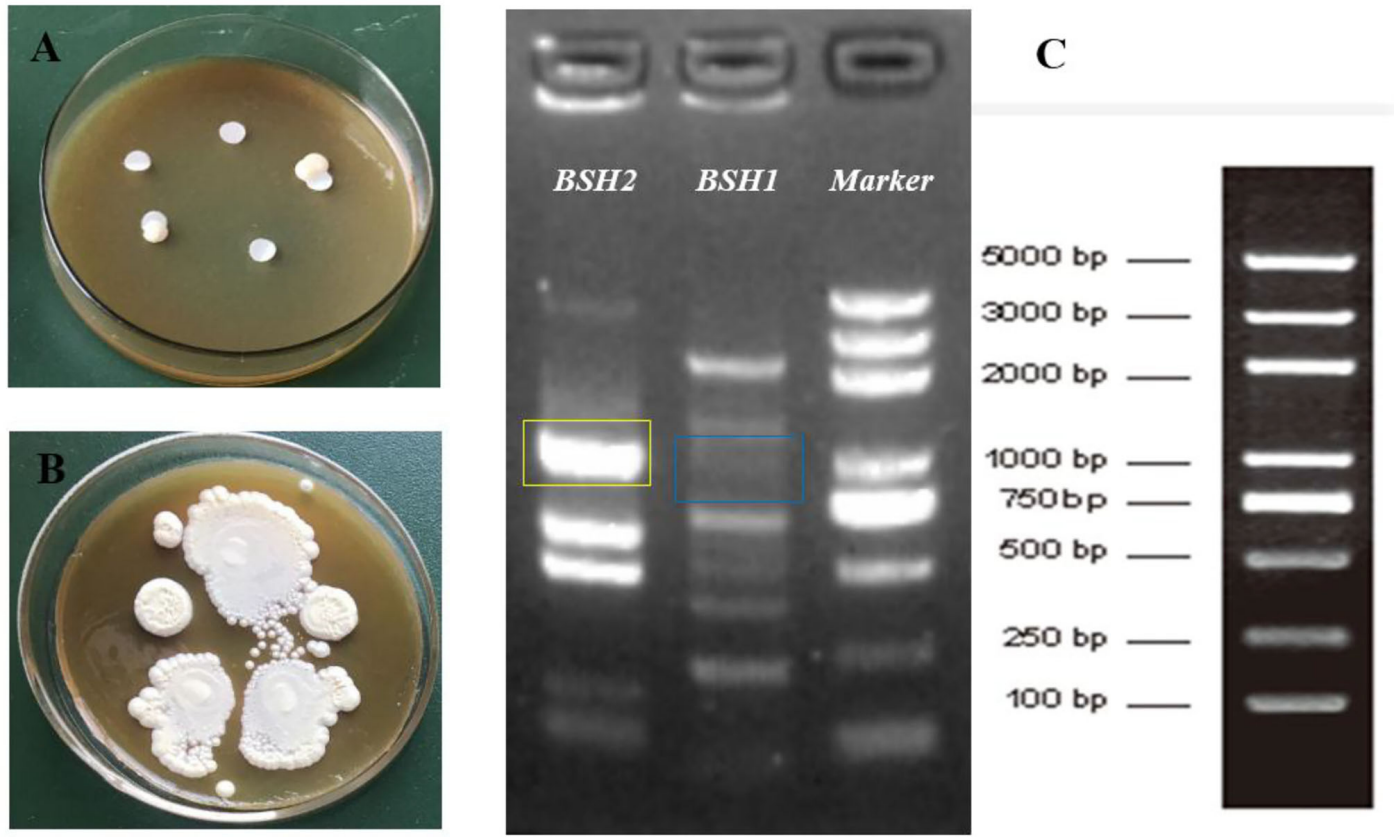
Identification of bile salt hydrolase (BSH) activity in *Lactobacillus delbrueckii*. Incubation on MRS plate containing bile salts for 12 h **(A)** and 72 h **(B)**, respectively. Amplification of BSH genes in *L. delbrueckii* **(C)** and target band sites for *BSH1* and *BSH2* gene were marked with blue and yellow box, respectively.

After incubation for 12 h, non-obvious BA precipitates appeared around the discs in the plate (Figure 1A); however, the opaque granular white colonies with silvery shine were observed after 72-h incubation (Figure 1B). PCR amplification of two designated genes showed that the BSH2 gene was identified, not BSH1, on the genome sequence of *L. delbrueckii* (Figure 1C).

### Serum Lipid Profiles

**Figure 2:**
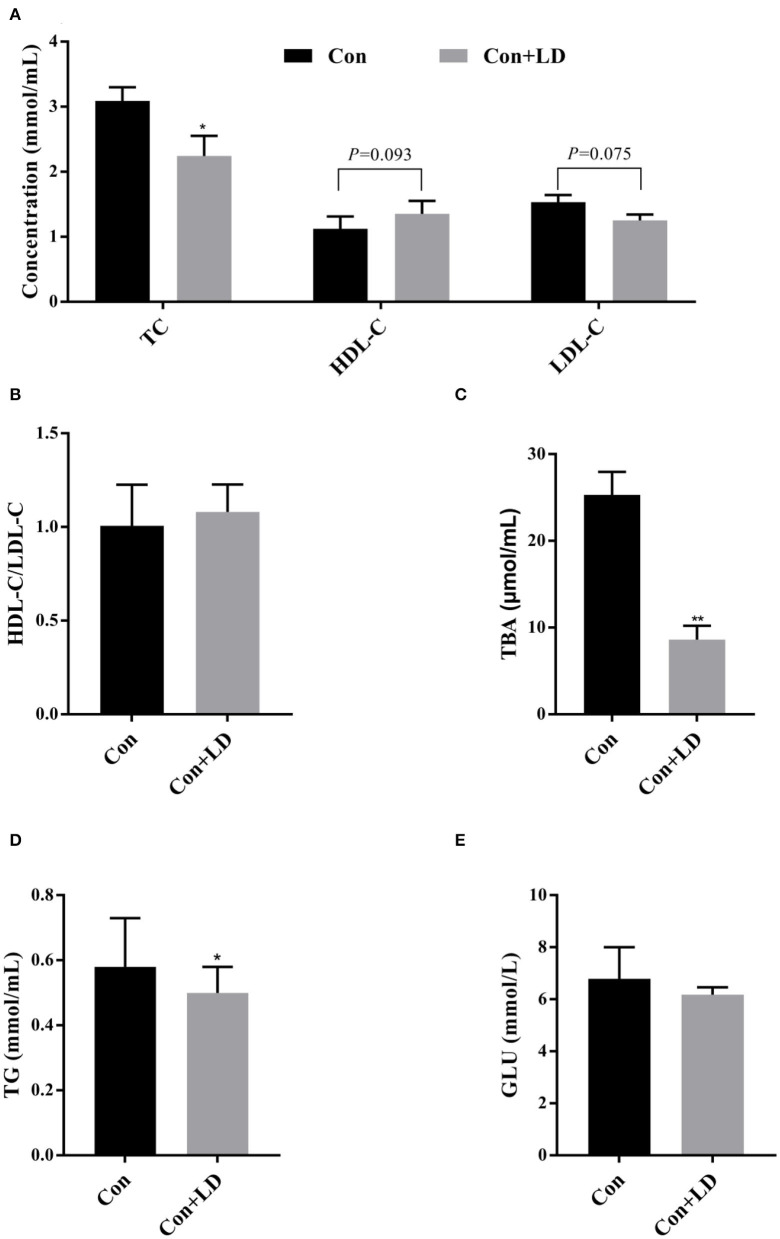
Effects of *Lactobacillus delbrueckii* on serum levels of TC, HDL-C, and LDL-C **(A)**; ratio of HDL-C/LDL-C **(B)**; TBA **(C)**; TG **(D)**; and GLU **(E)** in growing–finishing pig (*P < 0.05; **P < 0.01).

Serum TC, TBA, and TG contents in *L. delbrueckii–*fed pigs were found to be lower than the pigs in the Con group (*P* < 0.05; Figures 2A,C,D). *L. delbrueckii* treatment tended to reduce the concentration of serum LDL-C (*P* = 0.075) and elevate serum HDL-C (*P* = 0.093) level (Figure 2A). No significant changes in serum GLU and HDL-C/LDL-C contents were observed between two groups (*P* > 0.05, Figures 2B,E).

### Alterations in BA Profiles of Serum, Ileal Digesta, and Liver

**Figure 3:**
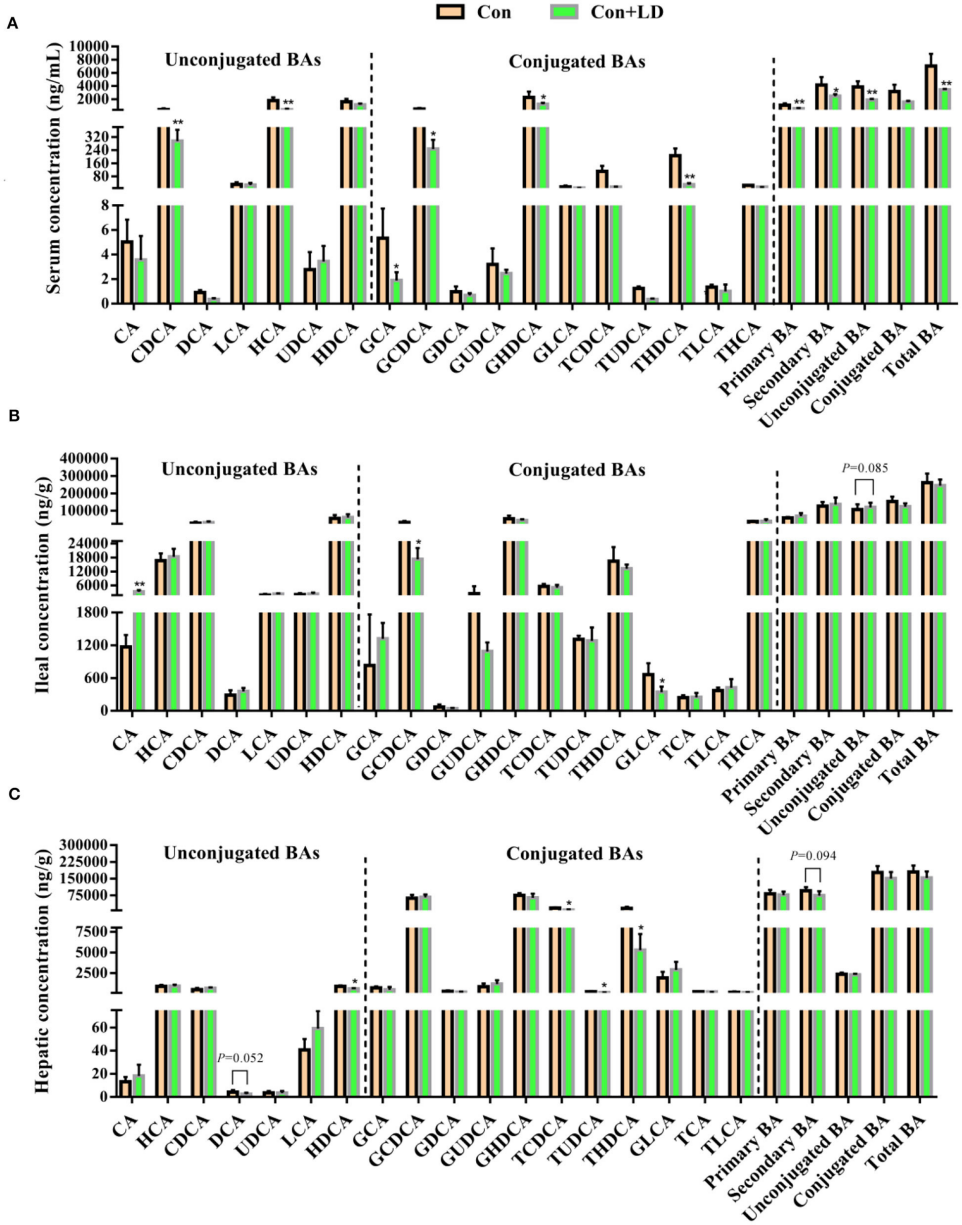
Bile acids profile in the serum **(A)**, ileal digesta **(B)**, and liver **(C)** of growing–finishing pigs (*P < 0.05; **P < 0.01).

Compared with the Con group, lower serum levels of CDCA, HCA, GCA, GCDCA, GHDCA, TUDCA, THDCA, primary BA, secondary BA, unconjugated BA, and total BA were found in the Con-LD group (*P* < 0.05, Figure 3A). Dietary addition of *L. delbrueckii* increased the ileal concentrations of CA and unconjugated BA (*P* = 0.085), but reduced GCDCA and GLCA (*P* < 0.05, Figure 3B). Hepatic concentrations of DCA (*P* = 0.052), HDCA, TCDCA, TUDCA, THDCA, and secondary BA (*P* = 0.094) in *L. delbrueckii–*fed pigs were decreased compared to the pigs in the Con group (*P* < 0.05, Figure 3C).

### Ileal Bacteria Composition

**Figure 4:**
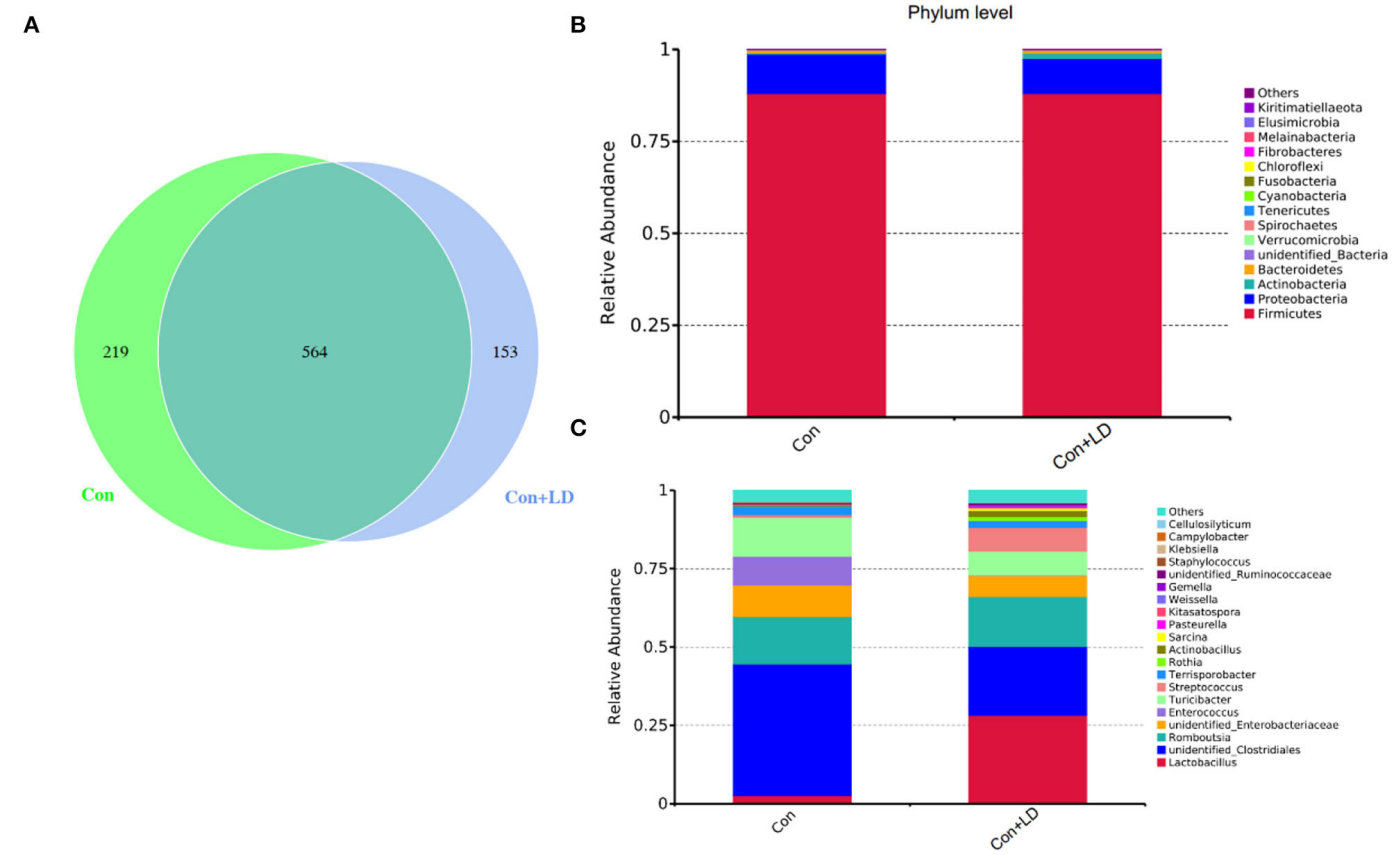
Ileal bacterial composition of growing–finishing pigs. Venn picture showed shared or unique OTUs between two groups **(A)**. Relative abundance of ileal microbiota at the phylum **(B)** or genus **(C)** level.

The Venn picture presented 546 shared OTUs between two groups, and there were 219 and 153 unique OTUs in the Con and Con + LD group, respectively (Figure 4A). The bacterial population was dominated by Firmicutes and Proteobacteria, with minor populations such as Actinobacteria and Bacteroidetes (Figure 4B). Administration of *L. delbrueckii* increased the abundance of Actinobacteria (*P* = 0.071), Spirochaetes (*P* = 0.070), and Kiritimatiellaeota (*P* = 0.029) and reduced the abundance of Melainabacteria (*P* = 0.091) and Elusimicrobia (*P* = 0.029). Down to the genus level, the higher abundance of *Lactobacillus* (*P* = 0.002) and lower abundance of *Clostridiales* (*P* = 0.031), *Ruminococcaceae* (*P* = 0.061), *Enterococcus* (*P* = 0.035), *Streptococcus* (*P* = 0.052), and *Rothia* (*P* = 0.049) were found (Figure 4C and Supplementary Table 1).

### BA and Cholesterol Transport, Biosynthesis, and Excretion

**Figure 5:**
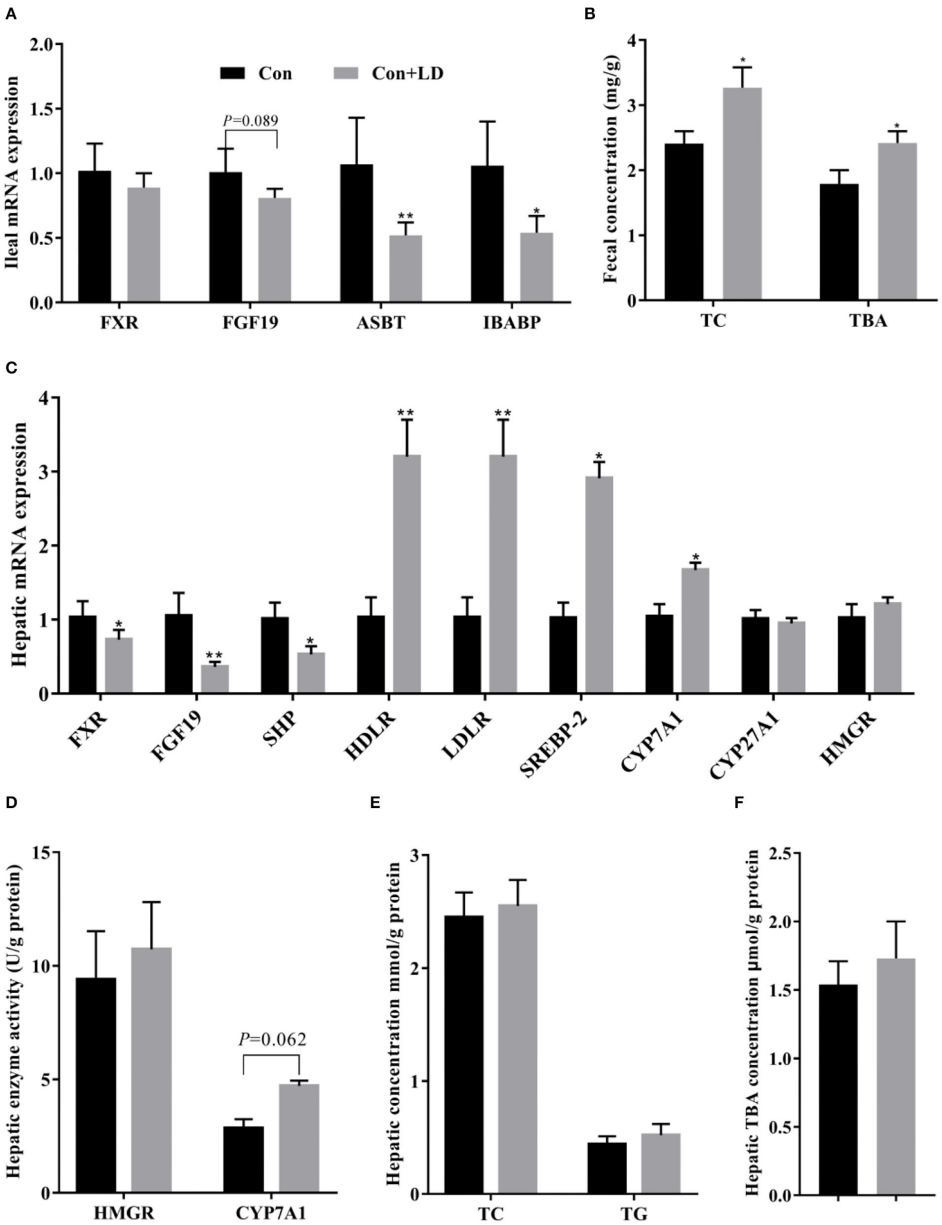
Bile acid and cholesterol transport, biosynthesis, and excretion of growing–finishing pigs. Bile acid receptors and transporters along the ileum **(A)**. Fecal TC and TBA excretion **(B)**. Bile acid metabolism-related genes in the liver **(C)**. Hepatic enzyme activity related to cholesterol and bile acid synthesis **(D)**. Hepatic TC and TG **(E)** and TBA **(F)** concentrations (*P < 0.05; **P < 0.01).

Administration of *L. delbrueckii* downregulated the gene expression of ileal FGF19 (*P* = 0.089), ASBT, and IBABP and enhanced fecal TC and TBA excretion (*P* < 0.05, Figures 5A,B). Hepatic gene expressions of FXR, FGF19, and SHP were reduced, but HDLR, LDLR, SREBP-2, and CYP7A1 were increased in the Con + LD group (*P* < 0.05, Figure 5C). Hepatic CYP7A1 activity tended to be greater in the *L. delbrueckii–*fed pigs than those in the Con group (*P* = 0.062, Figure 5D). No changes were found in hepatic concentrations of TC, TG, and TBA between two groups (*P* > 0.05, Figures 5E,F).

### Tissue TG and TC Deposition

**Figure 6:**
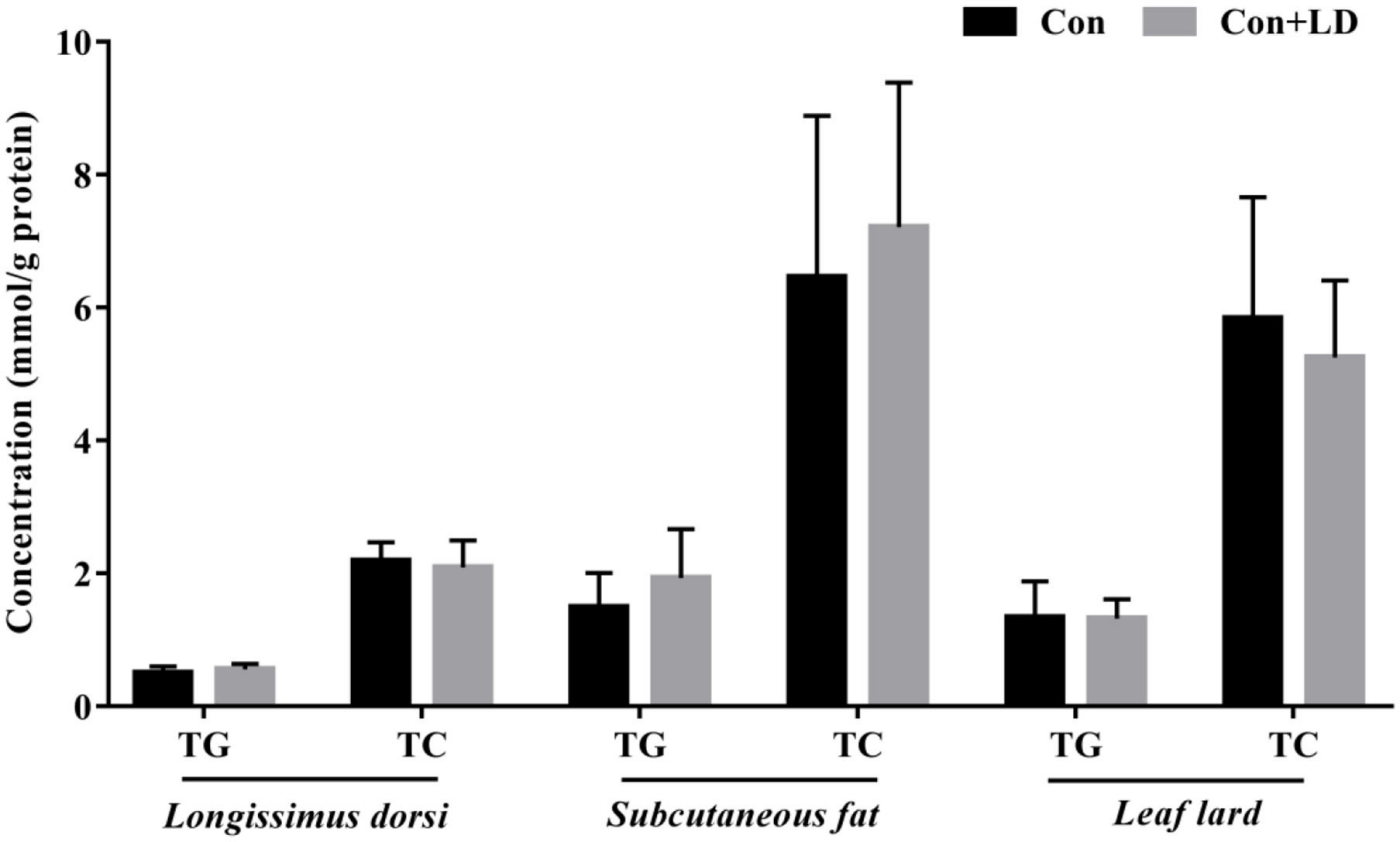
Concentrations of TG and TC in the selected tissues of growing–finishing pigs.

The concentrations of TG and TC in the longissimus dorsi, subcutaneous fat, and leaf lard had no differences between two groups (*P* > 0.05, Figure 6).

## Discussion

Fluctuation of blood lipids parameters can reflect the body's lipid metabolism and health status; chronically high serum TC and LDL-C levels are strongly associated with the increased risks of CVD (22, 23). TC and TG are the main components of blood lipids; lowering their concentrations can prevent hyperlipemia. Considerable researches have confirmed that consumption of *Lactobacillus* products reduced concentrations of serum cholesterol and improved lipid profiles (24–26). In the current study, dietary addition of *L. delbrueckii* decreased serum levels of TC, LDL-C, and TG of pigs. Our findings were again the proof of our previous reports (16) and also offered another evidence for cholesterol-lowering role of lactic acid bacteria in normal subject. The hypocholesterolemic effect of *L. delbrueckii* might provide a potential dietary manipulation way to prevent and improve hyperlipidemia.

Probiotics with BSH activity is hypothesized to be an important character in lowering serum cholesterol, which might be tightly related to the BSH genes on their genome (1, 4, 12, 22, 27). BSH activity has been characterized in *Lactobacillus, Bifidobacterium, Clostridium, Enterococcus*, and *Bacteroides* (12). *L. delbrueckii* tested in this study possessed the BSH2 gene identified by PCR amplification and exhibited a good BSH activity on the modified agar plate, demonstrating the strain was capable of bile salts deconjugation. BSH enzyme or activity is specific to the microbiota and is not present in eukaryotic cells, which is regarded as a crucial probiotic marker that help organisms resist toxic bile salt environment in the digestive tract and also an important colonization factor for gut bacteria (12, 28). *Lactobacillus* with BSH activity contribute to their survival and colonization in the gastrointestinal tract and exert a beneficial effect on host by regulating cholesterol and BA enterohepatic circulation (1, 4, 15, 29). Our results suggest that *L. delbrueckii* might own a good ability of intestinal survival and colonization and play a key role in regulating cholesterol and BA metabolism.

Intestinal microbiota and BA metabolism are mutually linked, enteric bacterial enzymes shape BA pool size and composition by mediating deconjugation and 7α-dehydroxylation of primary BAs (27). Liver cells synthesize primary BAs from cholesterol, mainly consisting of CA and CDCA in human, CA, α-/β-MCA in rodent and CA, HCA, and CDCA in pigs, and these BAs were conjugated with either glycine (G-BAs) or taurine (T-BAs) via their N-acyl amidate to increased solubility before secretion into intestine (30). Bile salt deconjugation is carried out by BSH, expressed in *Lactobacillus, Bifidobacterium, Clostridium*, and *Bacteroides* (9, 31). The genus *Lactobacillus* and its BSH activity could result in deconjugation of conjugated BAs (32). The conjugated BAs are very soluble, and most of them are reabsorbed in the ileum into enterohepatic circulation. In our study, *L. delbrueckii* administration obviously increased the ileal *Lactobacillus* abundance, indicating that ileal bacterial BA deconjugation might enhance. Interestingly, we found ileal concentrations of GCDCA, GLCA, and unconjugated BAs were decreased in the Con + LD group. Bacterial deconjugation of T-BA or G-BA can reduce serum cholesterol levels via amplifying the formation of new bile salts needed to replace those that have escaped enterohepatic cycle (26). Therefore, the potential mechanism of cholesterol reduction in *L. delbrueckii* might be the conversion of bile salt to free BA by improving ileal *Lactobacillus* abundance with BSH activity and interfered with BAs enterohepatic circulation.

In the intestine, bile salts play an important role in emulsifying lipids. Ileum is confirmed as the major site for BA reabsorption, and the highest expression of BA transporters and FGF19 was observed along the intestinal segment (10). Intestinal BA transporters play a vital role on the BA reabsorption process. ASBT and IBABP are important BA transporters engaging in BA active or passive transport. ASBT imports luminal BAs to the enterocytes where the BAs bind to IBABP and are transferred to the basement surface and then enter into portal vein with the help of MRP3 and OSTα/OSTβ transporters in the basolateral membrane (10). In the present study, ileal expression of *ASBT* and *IBABP* in *L. delbrueckii–*treated pigs was markedly down-regulated, indicating that less ileal BAs were reabsorbed after *L. delbrueckii* consumption.

The liver is the center of the synthesis and metabolism of cholesterol and BAs. Cholesterol *de novo* synthesis begins with acetyl-CoA, and HMGR is the rate-limiting enzyme responsible for catalyzing the conversion of HMG-CoA into mevalonic acid. Our results showed that administration of *L. delbrueckii* did not affect HMGR activity and mRNA expression, but upregulated hepatic *SREBP2, LDLR*, and *HDLR* expression. SREBP2 is a key nuclear transcription factor for regulating *LDLR* and *HMGR* target genes in charge of extrahepatic cholesterol uptake and endogenous cholesterol biosynthesis (33). Hepatic HDLR and LDLR are responsible for combining blood HDL-C and LDL-C to remove cholesterol, respectively. HDL-C carries cholesterol from peripheral tissues to liver; conversely, LDL-C transports hepatic cholesterol to peripheral tissues. Our observations implied *L. delbrueckii* treatment had no influence on hepatic cholesterol synthesis, but it might change its metabolism via hepatic clearance. The conversion of cholesterol to BAs is the main way to eliminate hepatic cholesterol, and CYP7A1 is the rate-limiting enzymes in the pathway (4). In the present study, hepatic *CYP7A1* expression was increased, and CYP7A1 activity also tended to rise in the Con + LD group, indicating that dietary *L. delbrueckii* might lower cholesterol via enhancing BAs biosynthesis.

Hepatic BA synthesis is negatively regulated by FXR signaling and FGF19 signaling (34). Ileal FXR activation contributes to FGF19 production, and then FGF19 translocates to the liver via hepatic portal vein where it binds to the FGFR4/β-Klotho complex and inhibits *CYP7A1* expression (9, 15). CYP7A1 is the rate-limiting enzyme in classic pathway for hepatic BA synthesis. Our results showed that ileal *FGF19* expression and hepatic *FXR* and *FGF19* expression were downregulated, but *CYP7A1* expression and CYP7A1 activity were increased by *L. delbrueckii* treatment, suggesting that this strain increased the conversion of cholesterol to BAs in the liver via suppressing FXR–FGF19 signaling and improving CYP7A1 activity. Additionally, reduction of hepatic FXR and SHP expression could also explain the increased CYP7A1 activity, because hepatic FXR stimulation resulted in SHP expression upregulation to inhabit CYP7A1 and CYP8B1 activity (3).

The homeostasis of BAs pool is maintained by enterohepatic cycle. Quantitative determination of BAs profiles via UPLC-MS analysis could reflect the enterohepatic circulation of BAs (35, 36). In our study, we observed great changes in BA composition of serum, ileal digesta, and liver, which might ascribe ileal microbiota modification with *L. delbrueckii*. Deconjugation of ileal bile salts causes less BAs to enter portal vein and return to liver, and unabsorbed BAs flow into hindgut and are excreted along with feces. Fecal BA excretion is almost equal to the hepatic synthesized BAs under the normal physiological condition (4, 37). Enhancement of BA synthesis using circulating cholesterol to restore the BA pool is an important manner for reduction of serum cholesterol (38, 39). In our study, dietary *L. delbrueckii* accelerated fecal TC and TBA output of pigs, which was closely associated to the decrease in serum TC and LDL-C. Reduction of serum cholesterol might lower cholesterol deposition in tissues; however, we found no alterations in TG and TC contents in longissimus dorsi, subcutaneous fat, and leaf lard, which implied that short-term *L. delbrueckii* treatment could not change tissue cholesterol deposition of growing–finishing pigs.

## Conclusions

**Figure 7:**
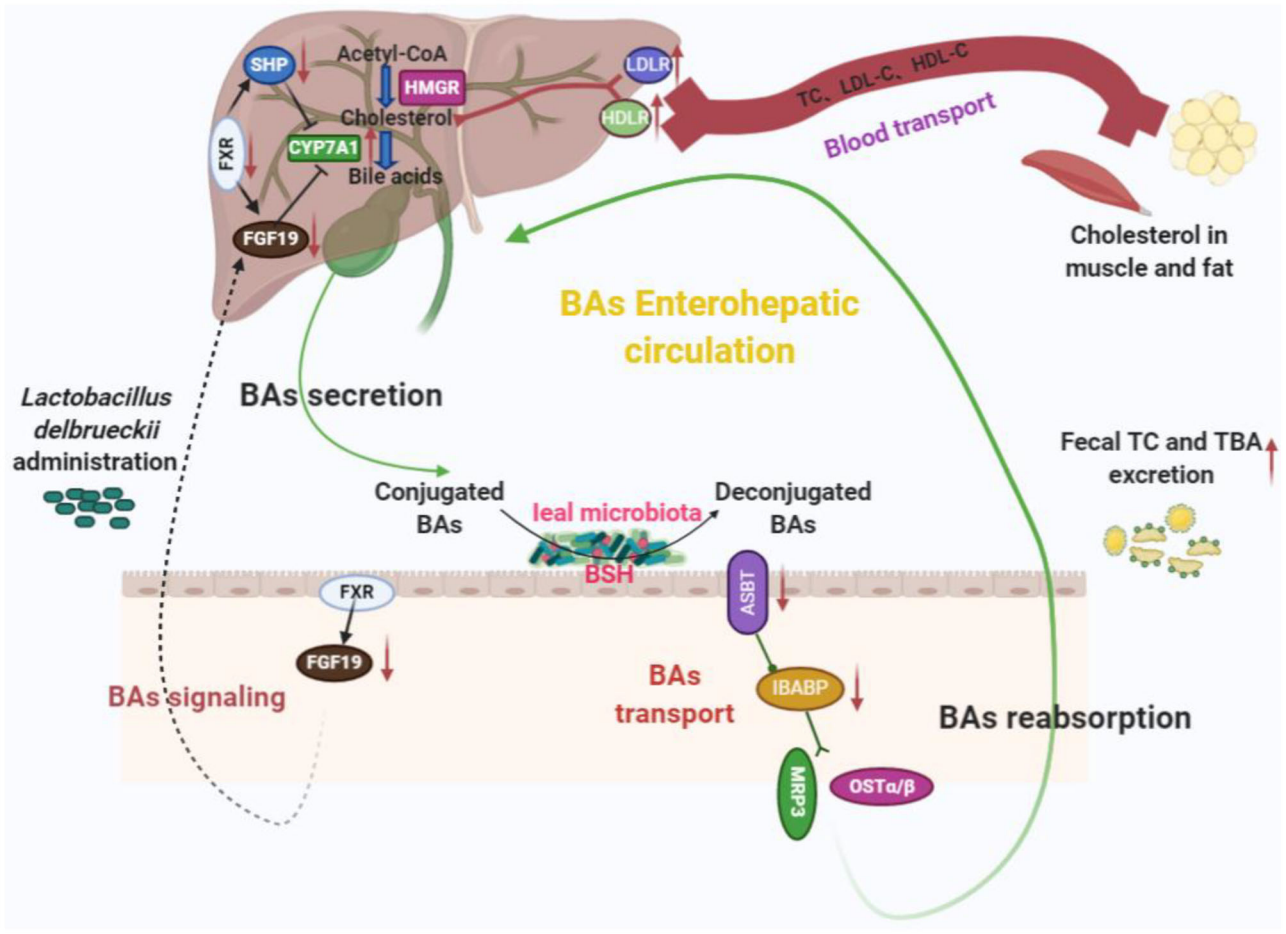
Potential cholesterol-lowering mechanisms of *Lactobacillus delbrueckii*–fed pigs. Ileal microbiota modification (mainly increased ileal *Lactobacillus* abundance) with *L. delbrueckii* administration led to bile salts deconjugation by BSH activity and facilitated fecal TBA and TC excretion. Meanwhile, less deconjugated BAs are reabsorbed into enterohepatic circulation and downregulate BA transporter expression (*ASBT* and *IBABP*) and promote BA synthesis via increasing the conversion of cholesterol to BA with the help of CYP7A1, a rate-limiting enzyme, in the liver. Additionally, ileal BA deconjugation by microbiota alteration affects BA signaling (FXR–FGF19 axis) to regulated BA synthesis. Enhancement of BA synthesis using circulating cholesterol (blood transport) to restore the bile acid pool.

Ileal microbiota modification induced by *L. delbrueckii* enhances BA deconjugation and fecal excretion in growing–finishing pigs. These events involved changes in ileal BA reabsorption, repression of the enterohepatic FXR–FGF19 axis, and increased hepatic BA neosynthesis (Figure 7).

## Data Availability Statement

The datasets presented in this study can be found in online repositories. The name of the repository and accession number are SRA and PRJNA670289, respectively.

## Ethics Statement

The animal study was reviewed and approved by the Animal Ethics Committee of Hunan Agricultural University. Written informed consent was obtained from the owners for the participation of their animals in this study.

## Author Contributions

GH, RL, and XH design the experiment. GH, RL, YY, and LW conducted the animal experiments. GH and RL wrote and revised the manuscript. XH, RL, and YY offered the experimental reagents and materials. GH, RL, and WP did experimental analysis, collected, and analyzed the data. GH and RL preparedthe figures and edited the manuscript. All authors reviewed the manuscript.

## Conflict of Interest

The authors declare that the research was conducted in the absence of any commercial or financial relationships that could be construed as a potential conflict of interest.
