# Supplementary material for: Lactobacillus delbrueckii Interfere With Bile Acid Enterohepatic Circulation to Regulate Cholesterol Metabolism of Growing–Finishing Pigs via Its Bile Salt Hydrolase Activity
Source: Front Nutr. 2020 Dec 11;7:617676. doi: 10.3389/fnut.2020.617676 (PMC7759492; doi:10.3389/fnut.2020.617676)
Supplement: Supplementary file 1 [file Table_1.DOC]

Supplementary table

Table 1 Relative abundance of phylum and genus level

| Items | Con | Con+LD | P-Value |
| --- | --- | --- | --- |
| Phylum level | | | |
| Firmicutes | 0.8803±0.0911 | 0.8802±0.2006 | 0.248 |
| Proteobacteria | 0.0959±0.0838 | 0.1069±0.2031 | 0.192 |
| Actinobacteria | 0.0139±0.0293 | 0.0036±0.0060 | 0.071 |
| Bacteroidetes | 0.0057±0.0034 | 0.0065±0.0025 | 0.508 |
| Unidentified_Bacteria | 0.0011±0.0020 | 0.0002±0.0002 | 0.043 |
| Verrucomicrobia | 0.0010±0.0020 | 0.0037±0.0003 | 0.147 |
| Spirochaetes | 0.0009±0.0009 | 0.0006±0.0003 | 0.070 |
| Tenericutes | 0.0007±0.0006 | 0.0007±0.0003 | 0.343 |
| Cyanobacteria | 0.0001±0.0001 | 0.0003±0.0004 | 0.111 |
| Fusobacteria | 0.0001±0.0002 | 0.0002±0.0002 | 0.203 |
| Chloroflexi | 0.0001±0.0001 | 0.0001±0.0001 | 0.173 |
| Fibrobacteres | 0.0001±0.0001 | 0.0001±0.0001 | 0.157 |
| Melainabacteria | 0.0001±0.0001 | 0.0001±0.0001 | 0.091 |
| Elusimicrobia | 0 | 0 | 0.029 |
| Kiritimatiellaeota | 0 | 0 | 0.029 |
| Others | 0.0002±0.0002 | 0.0002±0.0002 | 0.901 |
| Genus level | | | |
| *Lactobacillus* | 0.0251±0.0386 | 0.3056±0.3661 | 0.002 |
| *unidentified_Clostridiales* | 0.4211±0.2421 | 0.2192±0.2605 | 0.031 |
| *Romboutsia* | 0.1512±0.0682 | 0.0718±0.0821 | 0.708 |
| *unidentified_Enterobacteriaceae* | 0.1005±0.2036 | 0.0636±0.0934 | 0.242 |
| *Enterococcus* | 0.0920±0.1627 | 0.0010±0.0003 | 0.035 |
| *Turicibacter* | 0.1243±0.0853 | 0.0768±0.1003 | 0.604 |
| *Streptococcus* | 0.0080±0.0057 | 0.0468±0.0758 | 0.052 |
| *Terrisporobacter* | 0.0273±0.0262 | 0.0206±0.0261 | 0.721 |
| *Rothia* | 0.0010±0.0012 | 0.0004±0.0001 | 0.049 |
| *Actinobacillus* | 0.0043±0.0080 | 0.0095±0.0108 | 0.271 |
| *Sarcina* | 0.0004±0.0001 | 0.0084±0.0185 | 0.029 |
| *Pasteurella* | 0 | 0 | 0.033 |
| *Kitasatospora* | 0.0022±0.0044 | 0.0001±0.0001 | 0.032 |
| *Weissella* | 0.0001±0 | 0.0020±0.0041 | 0.033 |
| *Gemella* | 0 | 0.0018±0.0041 | 0.030 |
| *unidentified_Ruminococcaceae* | 0.0046±0.0015 | 0.0093±0.0094 | 0.061 |
| *Staphylococcus* | 0.0001±0 | 0.0009±0.0020 | 0.038 |
| *Klebsiella* | 0.0011±0.0015 | 0.0009±0.0011 | 0.527 |
| *Campylobacter* | 0.0001±0 | 0.0006±0.0014 | 0.055 |
| *Cellulosilyticum* | 0.0010±0.0013 | 0.0003±0.0003 | 0.047 |
| *Others* | 0.0223±0.0035 | 0.1041±0.1858 | 0.031 |

Table 2 Growth performance of growing-finishing pigs fed diets containing Lactobacillus delbrueckii

| Items | Con | Con+LD | P-Value |
| --- | --- | --- | --- |
| Initial body weight (IBW, kg) | 38.90±6.32 | 38.50±4.90 | 0.638 |
| Final body weight (FBW, kg) | 68.60±9.22 | 71.60±3.42 | 0.158 |
| Average daily gain (ADG, g) | 1060.71±183.24 | 1182.14±70.76 | 0.239 |
| Average daily feed intake (ADFI, g) | 2671.43±302.41 | 2889.29±123.96 | 0.248 |
| Feed to gain ratio (F/G) | 2.55±0.23 | 2.45±0.22 | 0.741 |
